# Comparison of the resection depth between endoscopic mucosal resection and underwater endoscopic mucosal resection for superficial non‐ampullary duodenal epithelial tumors: A retrospective study

**DOI:** 10.1002/deo2.70091

**Published:** 2025-03-19

**Authors:** Toshiki Horii, Yohei Harada, Gen Kitahara, Takuya Wada, Akinori Watanabe, Kenji Ishido, Hisatomo Ikehara, Chika Kusano

**Affiliations:** ^1^ Department of Gastroenterology Kitasato University of Medicine Kanagawa Japan; ^2^ Department of Pathology Kitasato University of Medicine Kanagawa Japan

**Keywords:** duodenum, endoscopic mucosal resection, superficial non‐ampullary duodenal epithelial tumor, underwater endoscopic mucosal resection, UWEMR

## Abstract

**Objectives:**

There is concern that underwater endoscopic mucosal resection (UWEMR) uses buoyancy to elevate the lesion for snare resection, resulting in a shallower resection depth than that in endoscopic mucosal resection (EMR). We aimed to compare conventional EMR and UWEMR in terms of resection depth.

**Methods:**

We retrospectively reviewed cases in which EMR or UWEMR was performed for superficial non‐ampullary duodenal epithelial tumors of ≤20 mm between April 2018 and February 2024. The endpoints were histological complete resection rate, en bloc resection rate, presence of muscularis mucosa and submucosa in the resection specimen, and submucosal index calculated from the resection specimen.

**Results:**

EMR was performed on 19 lesions and UWEMR was performed on 52 lesions. Histological complete and en bloc resection rates were not significantly different between EMR and UWEMR (57.9% and 63.5%, respectively, *p* = 0.78; 78.9% and 90.4%, respectively, *p* = 0.24). No significant differences were observed between EMR and UWEMR in the muscularis mucosa of the resected specimens (78.9% and 92.3%, respectively, *p* = 0.20). The presence of submucosa in resected specimens was encountered less often in EMR cases than in UWEMR cases (57.9% versus [vs.] 84.6%, *p* = 0.03). There were significant differences in the submucosal index in the resected specimens between EMR and UWEMR cases (median 0.15 [interquartile range 0–0.39] vs. 0.33 [0.17–0.57], *p* = 0.04).

**Conclusion:**

UWEMR potentially includes the submucosa within the specimen.

## INTRODUCTION

The detection rate of superficial non‐ampullary duodenal epithelial tumors (SNADETs) has increased recently due to the widespread use of endoscopy. Early therapeutic intervention is necessary because SNADETs are precancerous lesions.^1^, ^2^ Traditionally, endoscopic mucosal resection (EMR) has been widely used to treat SNADETs; however, the usefulness of underwater EMR (UWEMR), in which the lesion is buoyed and resected by filling the lumen with fluid without injection into the submucosa, has been reported.^3^

Compared with conventional EMR, UWEMR has a higher en bloc resection rate and less delayed bleeding^4^ because the buoyancy of water causes the lesion to protrude into the lumen, allowing for proper snaring, and because resection in the water reduces the burning effects on the duodenal wall. Therefore, UWEMR is beneficial as a treatment for duodenal lesions with anatomically thin intestinal walls and frequent complications after endoscopic resection.

Because UWEMR for SNADETs does not involve injection into the submucosa, it is unclear whether it can adequately resect the submucosa, and there are few reports on the depth of resection of UWEMR, with only a small number of cases studied.^5^ In endoscopic resection, it is advantageous to ensure sufficient resection depth to prevent local tumor recurrence by resecting the vertical margins. Additionally, vertical tumor remnants cannot be determined from endoscopic findings and can only be evaluated pathologically.

Here, we aimed to compare conventional EMR and UWEMR in terms of resection depth.

## METHODS

### Ethics statements

This study was conducted in accordance with the principles of the Declaration of Helsinki. The Institutional Review Board at Kitasato University Hospital approved the study protocol on April 25, 2024 (approval number: B23‐179). Patients provided informed consent through an opt‐out option on the hospital's website.

### Study design and patients

In this retrospective, single‐center study, data were obtained from the patients’ medical records at Kitasato University Hospital. Patients with SNADETs diagnosed with adenomas or adenocarcinomas of ≤20 mm by endoscopy who underwent EMR or UWEMR between April 2018 and February 2024 were included. This study had no exclusion criteria, so all consecutive cases within the study period were included.

### EMR and UWEMR procedures

At Kitasato University Hospital, UWEMR has been implemented since April 2020. Prior to April 2020, EMR was performed for all cases; however, for cases after April 2020, the choice between EMR and UWEMR was left to the endoscopists’ discretion. All endoscopic procedures were performed with patients under sedation with midazolam and pethidine, and the Japan Gastroenterological Endoscopy Society criteria for withdrawal of antithrombotic agents were met.^6^, ^7^ A standard single‐channel endoscope (GIF‐Q260J; Olympus Optical or GIF‐H290T; Olympus Optical) was used for endoscopic resection. A 15‐mm electrosurgical polypectomy snare Snaremaster Plus; Olympus Optical) and the VIO 300D or VIO 3 system (ERBE Elektromedizin GmbH) were used as electrical currents for resection. The treatment was started with patients in the left lateral position; however, when UWEMR was performed, the patient's position was changed as needed to fill the lumen with fluid. For each treatment, a scope was inserted into the duodenum and ensured a suitable field of view for treatment and diagnosis of the lesion area. Before resection, a 10% glycerin solution (Glyceol; Chugai Pharmaceutical Co.) with indigo carmine was injected into the submucosa of patients in the EMR group. In the UWEMR group, saline was injected into the duodenum until the lumen was filled prior to resection. In both treatments, snaring was performed around the lesion to include the normal mucosa before resection, after which the specimen was promptly collected, and the ulcer was closed using endoscopic clips for mucosal defects. In the case of UWEMR resection, the endoscopist decided whether to suture the ulcer under water immersion using an endoscopic clip. The obtained specimens were stored in a 10% formalin solution and diagnosed by a pathologist. Pathological evaluation was also performed, and upper gastrointestinal endoscopy was performed 3–6 months later to evaluate local recurrence in cases where the resection margins were unclear.

### Outcome measures

**FIGURE 1:**
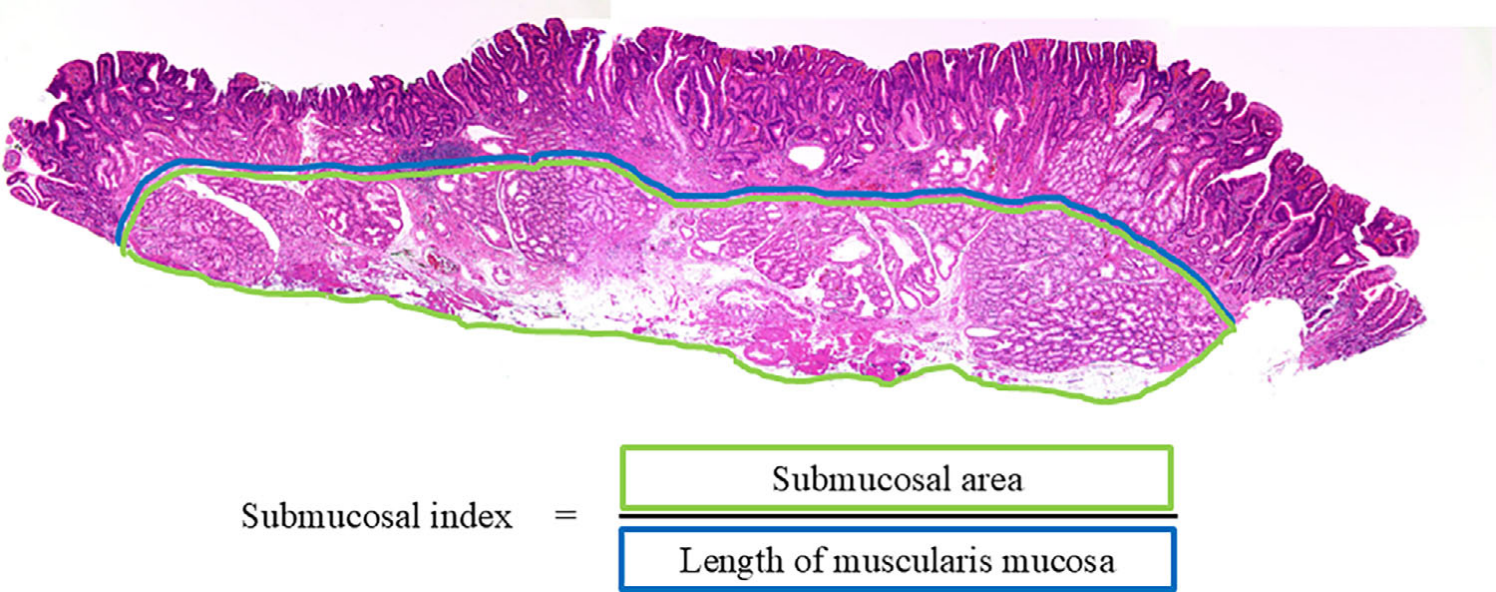
Pathological image evaluation. The length of the muscularis mucosa and thicknesses of the submucosa and the submucosal area were measured using NDP.view2. The submucosal index was defined as the submucosal area divided by the length of the muscularis mucosa.

The primary outcome was the resection depth in the EMR and UWEMR groups. The resection depth was evaluated on the basis of the presence of the muscularis mucosae and submucosa at the center of the lesion, thickness of the submucosa, submucosal area, and submucosal index. Evaluation of the resection depth was performed histologically using the largest section of the resected specimen. The presence of the muscularis mucosae and submucosa was evaluated on the basis of their attachment at the center of the lesion, and the thickness of the submucosa was measured as the distance from the muscularis mucosae to the submucosa at the center of the lesion. The submucosal index was defined as the area of the submucosa of the largest section of each excised specimen divided by the length of the muscularis mucosa, according to previous reports^5^, ^8^ (Figure 1). The area of the submucosa and the length of the muscularis mucosa were measured using image processing software (NDP.view2; Hamamatsu Photonics K.K.). The secondary outcomes were the histological complete resection rate, en bloc resection rate, clinical success rate, level of endoscopist experience, thickness of the submucosa of the resected specimen, procedure time, closure of the mucosal defect, exposure of muscle layers, and adverse events. Complete histological resection was defined as en bloc endoscopic resection of the entire lesion with histologically tumor‐free margins, both horizontally and vertically. Clinical success was defined as no local recurrence on upper gastrointestinal endoscopy after 3–6 months of follow‐up in cases of histologically diagnosed complete resection or in cases where it was histologically unclear whether the resection was complete. In classifying the level of endoscopist experience, we defined experienced endoscopists as those having experience with treating at least 10 cases of SNADETs. Procedure time was defined as the time from injection into the submucosa to the end of resection of the lesion in EMR cases and from the beginning of filling the duodenum with fluid to the end of resection in UWEMR cases. Immediate bleeding was defined as persistent bleeding lasting >1 min after resection or eruptive bleeding that occurred immediately after resection. Delayed bleeding was defined as any symptomatic hematemesis or tarry stool for which endoscopy was performed. Immediate perforation was defined as perforation confirmed by endoscopy. Delayed perforation was defined as the presence of abdominal pain and free air on imaging.

### Statistical analyses

Differences between categorical variables were examined using Fisher's exact probability and chi‐square tests. Differences between continuous variables were examined using the Mann–Whitney *U* test. Statistical significance was set at *p* < 0.05. All statistical analyses were performed using EZR (version 1.54).^9^

## RESULTS

### Patients’ baseline characteristics

Table 1 shows the patient characteristics. There were no significant differences in age or sex between the EMR and UWEMR groups. Significantly more patients in the EMR group than in the UWEMR group had a history of antiplatelet agent use.

**TABLE 1:** Patients’ baseline characteristics.

	EMR (*n* = 19)	UWEMR (*n* = 52)	*p*‐value
Age (years), SD	61.3 +14.8	61.7 +14.3	0.93
Sex, male, *n* (%)	14 (73.7)	37 (71.2)	0.78
Antithrombotic agents, *n* (%)			
Antiplatelet agents	4 (21.1)	2 (38.5)	0.04
DOACs	0 (0)	1 (1.9)	1.00

Abbreviations: DOACs, direct oral anticoagulants; EMR, endoscopic mucosal resection; UWEMR, underwater endoscopic mucosal resection.

### Characteristics of the resected lesions

Table 2 shows the characteristics of the resected lesions. There were no significant differences in lesion size, morphology, and histology between the groups. However, lesions were significantly more common in the second portion of the UWEMR group than in the EMR group.

**TABLE 2:** Lesion characteristics.

	EMR (*n* = 19)	UWEMR (*n* = 52)	*p*‐value
Size (mm), median (IQR)	10 (5.5–10)	7(5–9.25)	0.11
Location, *n* (%)			
Bulb	9 (47.4)	8 (15.4)	0.01
Second portion	10 (52.6)	44 (84.6)	
Morphology, *n* (%)			
Protruded	6 (31.6)	5 (9.6)	0.06
Flat	13 (68.4)	47 (90.4)	
Histology, *n* (%)			
Adenoma	18 (94.7)	50 (96.2)	1.00
Adenocarcinoma	1 (5.3)	2 (3.8)	

Abbreviations: EMR, endoscopic mucosal resection; IQR, interquartile range; UWEMR, underwater endoscopic mucosal resection.

### Results of both treatments

Table 3 presents the results of both treatments. There was no significant difference in the level of endoscopist experience between endoscopists who performed the resection for both treatments. There were no significant differences in the complete histological resection and en bloc resection rates between the EMR and UWEMR groups. Follow‐up was not performed in two patients who underwent UWEMR with unclear histological evaluations of the resection margins of the resected specimens. One case of local recurrence was observed in a patient in whom the histological evaluation of the resection margins of the EMR specimen was unclear. No local recurrence was observed in a case in which the histological evaluation of the resection margins of the UWEMR specimen was unclear. The clinical success rate and percentage of specimens containing the muscularis mucosa were not significantly different between the EMR and UWEMR groups. The presence of submucosa in resected specimens was encountered significantly less frequently in the EMR group than in the UWEMR group (57.9% versus 84.6%, *p* = 0.03). In the EMR and UWEMR groups, there were no significant differences in the median thickness of the submucosa and median submucosal area. However, there were significant differences in the median submucosal index of the resected specimens between the EMR and UWEMR groups (0.15 and 0.33, respectively, *p* = 0.04). There was also no significant difference in the median procedure time between the EMR and UWEMR groups. Exposure of the muscle layer at the ulcer base due to resection was not observed with either treatment. The only adverse event was a case of immediate bleeding; however, there was no significant difference between the EMR and UWEMR groups.

**TABLE 3:** Results of both treatments.

	EMR (*n* = 19)	UWEMR (*n* = 52)	*p*‐value
Histological complete resection, *n* (%)	11 (57.9)	33 (63.5)	0.78
En bloc resection, *n* (%)	15 (78.9)	47 (90.4)	0.24
Horizontal margin, *n* (%)			
HM0	12 (63.2)	33 (63.5)	0.35
HMX	3 (15.8)	14 (26.9)	
HM1	4 (21.1)	5 (9.6)	
Vertical margin, *n* (%)			
VM0	14 (73.6)	45 (86.5)	0.31
VMX	5 (26.3)	6 (11.5)	
VM1	0 (0)	1 (1.9)	
Clinical success, *n* (%)	14 (73.7)	45 (86.5)	0.28
Level of endoscopist experience, *n* (%)			
Experienced	19 (100)	45 (86.5)	0.18
Trainee	0 (0)	7 (13.5)	
Specimen containing the muscularis mucosa, *n* (%)	15 (78.9)	48 (92.3)	0.20
Specimen containing the submucosa, *n* (%)	11 (57.9)	44 (84.6)	0.03
Thickness of the submucosa, mm, median (IQR)	0.20 (0–0.98)	0.70 (0.40–1.11)	0.13
Submucosal area, mm^2^, median (IQR)	1.75 (0–3.89)	3.34 (1.72–5.71)	0.07
Submucosal index, median (IQR)	0.15 (0–0.39)	0.33 (0.17–0.57)	0.04
Procedure time (sec), median (IQR)	300.0 (203.0–491.5)	290.0 (218.3–436.3)	0.83
Closure of the mucosa defect, *n* (%)	18 (94.7)	52 (100)	0.27
Exposure of muscle layers, *n* (%)	0 (0)	0 (0)	–
Adverse events, *n* (%)			
Immediate bleeding	3 (15.8)	4 (7.7)	0.38
Delayed bleeding	0 (0)	0 (0)	–
Immediate perforation	0 (0)	0 (0)	–
Delayed perforation	0 (0)	0 (0)	–

Abbreviations: IQR, interquartile range; ‐, not applicable.

### Resection depth by the resection site

Table 4 shows the resection depth by the resection site. For the SNADET resection specimens of the bulb, no significant difference was observed between the EMR and UWEMR groups in the proportion of resected specimens containing the muscularis mucosae or the submucosa. These specimens also showed no significant difference in median submucosal thickness and median submucosal area between the groups. Further, there were no significant differences in the median submucosal index in the resected specimens of the bulb between the groups. For SNADET resection specimens of the second portion, no significant difference was observed between the groups in the proportion of resected specimens containing the muscularis mucosae or the submucosa. These specimens also showed no significant difference in median submucosal thickness and median submucosal area between the groups. Additionally, there were no significant differences in the median submucosal index in the resected specimens of the bulb between the groups.

**TABLE 4:** Resection depth by the resection site.

Bulb			
	EMR (*n* = 9)	UWEMR (*n* = 8)	*p*‐value
Specimen containing the muscularis mucosa, *n* (%)	8 (88.9)	7 (87.5)	1.00
Specimen containing the submucosa, *n* (%)	5 (55.6)	7 (87.5)	0.29
Thickness of the submucosa, µm, median (IQR)	0.20 (0–0.96)	1.15 (0.61–1.70)	0.13
Submucosal area, mm^2^, median (IQR)	2.13 (0.77–3.92)	6.89 (2.08–7.74)	0.29
Submucosal index, median (IQR)	0.26 (0–0.85)	0.89 (0.47–0.98)	0.26

Second portion			
	EMR (*n* = 10)	UWEMR (*n* = 44)	*p*‐value
Specimen containing the muscularis mucosa, *n* (%)	7 (70.0)	41 (93.2)	0.07
Specimen containing the submucosa, *n* (%)	6 (60.0)	37 (84.1)	0.19
Thickness of the submucosa, mm, median (IQR)	0.24 (0–0.91)	0.62 (0.36–0.98)	0.27
Submucosal area, mm^2^, median (IQR)	1.49 (0–3.86)	3.27 (1.60–4.53)	0.13
Submucosal index, median (IQR)	0.27 (0–0.62)	0.58 (0.39–0.72)	0.17

Abbreviations: IQR, interquartile range.

## DISCUSSION

Our findings highlight the potential advantages of UWEMR compared with conventional EMR in achieving deeper resection. The strength of our study is that we evaluated pathological specimens from >50 SNADETs treated with UWEMR, whereas previous reports on the depth of resection of EMR and UWEMR for SNADETs focused on a small number of cases. We found that the rate of the presence of the submucosa was significantly higher in the UWEMR group than in the EMR group, and the submucosa within the resected specimens was significantly thicker.

The major differences between the EMR and UWEMR techniques are the injection into the submucosa and the filling of the lumen with fluid. We expected that UWEMR, which requires the lumen to be filled with water, would require more time than conventional EMR; however, the procedure time was not significantly different. Water jet function and changing the patient's body position may have been effective in reducing the time required to fill the duodenal lumen with fluid. Additionally, submucosal injections can sometimes cause unexpected bleeding, which might have been one of the reasons why there was no significant difference in the procedure time.

Duodenal tumors are rare, and whether endoscopic treatment is curative remains controversial. Sakamoto et al. reported no lymph node metastasis in cases of intramucosal carcinoma of the duodenum.^10^ In contrast, lymph node metastasis has been reported in 42%–57% of submucosal invasive carcinomas requiring surgical intervention.^10^, ^11^ Therefore, histological evaluation of suspected duodenal cancer lesions is necessary to determine the treatment course after endoscopic resection. In our study, the resection depth achieved with UWEMR was significantly greater than that achieved with conventional EMR using submucosal injection. We observed a difference of approximately 0.2 in the submucosal index. Given the high prevalence of lymph node metastases in duodenal cancer with submucosal invasion, this difference is considered to have limited clinical significance. A comparison of the first quartile of the submucosal index between the two treatments suggests that UWEMR is a valuable resection method for determining post‐resection treatment strategies. Previous studies by Toyosawa et al. and Miyazaki et al. showed that UWEMR allows for the resection of the submucosal layer compared with other resection methods such as CSP and HSP, which do not involve submucosal injection, consistent with the present findings.^8^, ^12^ These reports suggest that the resection depth of UWEMR is deeper than that of other methods. EMR involves injections into the submucosa with the goal of achieving sufficient resection depth. However, the tense mucosa containing the lesion due to injection into the submucosa may slide over the mucosa when the snare is tightened, causing it to move from the planned resection line to the lesion side, resulting in cases where the submucosa is not resected or the submucosa to be resected is thin. Since UWEMR is only elevated by buoyancy and there is no tension on the mucosa, it is thought that the snare is sufficiently tightened to maintain a resection line that includes the submucosa and allows resection that includes the submucosa. Regarding the resection depth for SNADETs, studies have reported no significant difference between EMR and UWEMR, which is contrary to our findings.^5^ The difference between this previous study and ours likely lies in the handling of the resected specimens. Toya et al. fixed the resected specimens in formalin after pinning them to a corkboard. Consequently, the length of the muscularis mucosae of their specimens may have become longer than that of our specimens, potentially leading to a difference in the submucosal index.

Anatomically, the duodenal bulb is characterized by a well‐developed presence of Brunner glands. Consequently, in the duodenal bulb, the elevation of lesions following submucosal injection may be poor. Therefore, we analyzed the resection depth by dividing the sites into the bulb and the second portion of the duodenum and found no significant difference in the resection depth between EMR and UWEMR for SNADET resection specimens of the bulb. Brunner glands are primarily located in the submucosa but may extend into the mucosal layer.^13^, ^14^ This suggests that even under a water immersion environment, the mucosal layer in the duodenal bulb may inherently be less prone to elevation due to buoyancy.

There have been multiple reports on the rate of complete histologic resection by UWEMR and EMR for SNADET.^4^, ^15^ Here, the histological complete resection rate with UWEMR was 63.5%, which was not significantly different from that with conventional EMR; however, previous studies have reported histological complete resection rates of 50.8%–75.8% with UWEMR, which are almost similar.^4^, ^15^, ^16^, ^17^, ^18^ Bhogal et al. defined clinical success as the absence of local recurrence on follow‐up examination after UWEMR and reported a rate of 89.9%, which was similar to that in the present study.^19^ Based on these facts, our results using UWEMR can be considered standard, but further improvement in the rate of complete histologic resection requires addressing cases in which the evaluation of the ablative margin is unclear. The dissociation between complete histological resection and clinical success is affected by the burning effect of electrical currents on specimens, which makes the horizontal and vertical margins difficult to assess. In this study, UWEMR was performed by filling the lumen with saline solution for resection. Because saline has higher conductivity than air, more energy is required to resect the lesion.^20^ Therefore, specimens resected using UWEMR may experience a severe burning effect. This suggests that snaring around the lesion with a normal margin that considers the burning effect is necessary to improve the rate of complete histological resection.

This study has some limitations. This was a retrospective, single‐center study, and there was a significant difference between the number of EMR and UWEMR procedures performed. Additionally, there were differences in the location of the lesions. Lastly, this study evaluated local recurrence at 3–6 months after treatment, which may be an early follow‐up time. Hence, prospective comparative studies and evaluation of local recurrence in the long term are warranted.

In conclusion, UWEMR is a promising resection technique that allows for deeper submucosal inclusion, which may have implications for optimizing treatment strategies for duodenal tumors. Further studies are needed to confirm its long‐term clinical efficacy.

## CONFLICT OF INTEREST STATEMENT

Toshiki Horii, Yohei Harada, Gen Kitahara, Takuya Wada, Akinori Watanabe, and Kenji Ishido declare no conflict of interest. Hisatomo Ikehara received honoraria for his lectures from FUJIFILM Co., Olympus Co., Otsuka Pharmaceutical Co., AstraZeneca K. K., Takeda Pharmaceutical Co., AI Medical Service Inc., 3‐D Matrix Ltd., and Mundiphama K.K. Chika Kusano received honoraria for her lectures from FIJIFILM Medical Co., Olympus Co., Takeda Pharmaceutical Co., and Otsuka Pharmaceutical Co.

## ETHICS STATEMENT

Approval of the research protocol by an Institutional Reviewer Board: This study was conducted in accordance with the principles of the Declaration of Helsinki. The Institutional Review Board at Kitasato University Hospital approved the study protocol on April 25, 2024 (approval number: B23‐179).

## PATIENT CONSENT STATEMENT

Written consent was not required because patients provided informed consent through an opt‐out option on the hospital's website.

## CLINICAL TRIAL REGISTRATION

N/A.

## Data Availability

The data that support the findings of this study are not publicly available due to their containing information that could compromise the privacy of research participants but are available from the corresponding author Chika Kusano upon reasonable request.
